# QuickStats

**Published:** 2015-01-09

**Authors:** FROM THE NATIONAL CENTER FOR HEALTH STATISTICS

**Figure:**
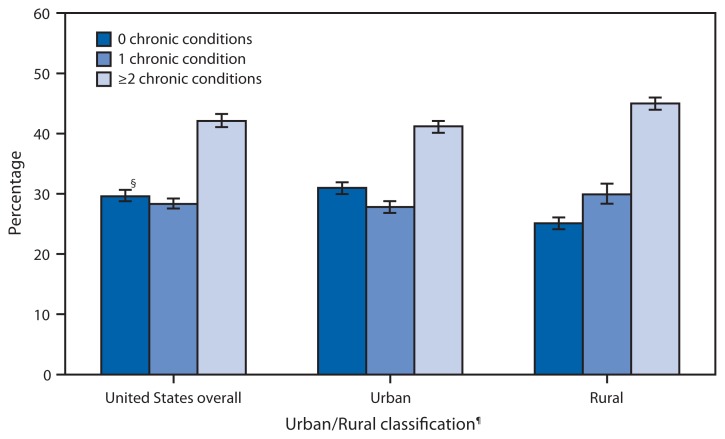
Percentage of Adults Aged ≥45 Years with Selected Diagnosed Chronic Conditions,* by Number of Conditions and Urban/Rural Classification — National Health Interview Survey, 2013^†^ * The 10 selected chronic conditions are hypertension, coronary heart disease, stroke, diabetes, cancer, arthritis, hepatitis, chronic obstructive pulmonary disease (COPD), weak or failing kidneys during the past 12 months, currently having asthma. COPD was defined as having emphysema or chronic bronchitis during the past 12 months, or both. Unless a timeframe is otherwise noted, chronic conditions are based on ever being told by a doctor or other health professional that the respondent has the condition. ^†^ Estimates are based on household interviews of a sample of the noninstitutionalized U.S. civilian population and are derived from the National Health Interview Survey sample adult component. ^§^ 95% confidence interval. ^¶^ The 2000 U.S. Census definition was used in this classification, where adults residing in a core of census tracts and/or census blocks with a population of 2,500 persons or more were considered living in an urban area. Adults living in census tracts and/or census blocks with fewer than 2,500 were considered living in a rural area.

In 2013, 29.6% of U.S. adults aged ≥45 years had none of the 10 selected diagnosed chronic conditions, 28.3% had one condition, and 42.1% had multiple (two or more) conditions. A higher percentage of adults aged ≥45 years living in rural areas had multiple chronic conditions compared with adults in urban areas (45.0% versus 41.2%), whereas a lower percentage had none (25.1% versus 31.0%).

**Source:** National Health Interview Survey, 2013 data. Available at http://www.cdc.gov/nchs/nhis.htm.

**Reported by:** Brian W. Ward, PhD, ijz8@cdc.gov, 301-458-4568; Jeannine S. Schiller, MPH.

